# IL-2 produced by HBV-specific T cells as a biomarker of viral control and predictor of response to PD-1 therapy across clinical phases of chronic hepatitis B

**DOI:** 10.1097/HC9.0000000000000337

**Published:** 2023-12-07

**Authors:** Conan Chua, Loghman Salimzadeh, Ann T. Ma, Oyedele A. Adeyi, Hobin Seo, Giselle M. Boukhaled, Aman Mehrotra, Anjali Patel, Sara Ferrando-Martinez, Scott H. Robbins, Danie La, David Wong, Harry L.A. Janssen, David G. Brooks, Jordan J. Feld, Adam J. Gehring

**Affiliations:** 1 Institute of Medical Sciences, University of Toronto, Toronto, Ontario, Canada; 2 Toronto Centre for Liver Disease, Toronto General Research Institute, University Health Network, Toronto, Ontario, Canada; 3 Liver Unit, Hospital Clínic de Barcelona, Barcelona, Spain; 4 Department of Laboratory Medicine and Pathology, University of Minnesota Medical School, Minneapolis, Minnesota, USA; 5 Princess Margaret Cancer Centre, University Health Network, Toronto, Ontario, Canada; 6 Microbial Sciences, Biopharmaceuticals R&D, AstraZeneca, Gaithersburg, Maryland, USA; 7 Late Stage Oncology Development, Oncology R&D, AstraZeneca, Gaithersburg, Maryland, USA; 8 Department of Immunology, University of Toronto, Toronto, Ontario, Canada

## Abstract

**Background:**

There are no immunological biomarkers that predict control of chronic hepatitis B (CHB). The lack of immune biomarkers raises concerns for therapies targeting PD-1/PD-L1 because they have the potential for immune-related adverse events. Defining specific immune functions associated with control of HBV replication could identify patients likely to respond to anti-PD-1/PD-L1 therapies and achieve a durable functional cure.

**Methods:**

We enrolled immunotolerant, HBeAg+ immune-active (IA+), HBeAg− immune-active (IA−), inactive carriers, and functionally cured patients to test *ex vivo* PD-1 blockade on HBV-specific T cell functionality. Peripheral blood mononuclear cells were stimulated with overlapping peptides covering HBV proteins +/−α-PD-1 blockade. Functional T cells were measured using a 2-color FluoroSpot assay for interferon-γ and IL-2. *Ex vivo* functional restoration was compared to the interferon response capacity assay, which predicts overall survival in cancer patients receiving checkpoint inhibitors.

**Results:**

*Ex vivo* interferon-γ+ responses did not differ across clinical phases. IL-2+ responses were significantly higher in patients with better viral control and preferentially restored with PD-1 blockade. Inactive carrier patients displayed the greatest increase in IL-2 production, which was dominated by CD4 T cell and response to the HBcAg. The interferon response capacity assay significantly correlated with the degree of HBV-specific T cell restoration.

**Conclusions:**

IL-2 production was associated with better HBV control and superior to interferon-γ as a marker of T cell restoration following ex vivo PD-1 blockade. Our study suggests that responsiveness to ex vivo PD-1 blockade, or the interferon response capacity assay, may support stratification for α-PD-1 therapies.

## INTRODUCTION

Functional restoration of exhausted HBV-specific T cells is a primary goal of new therapies, given their critical role in controlling and clearing HBV infection.^1–3^ HBV-infected chimpanzee studies demonstrated that global deletion of either CD4 or CD8 T cells led to a severely delayed capacity to control HBV, indicating that T cells are the main mediators of HBV viral control.^3,4^ However, T cell analysis in the context of viral control has focused on the magnitude of response rather than specific T cell functions. HBV-specific T cells robustly expand in patients successfully resolving acute infection,^5–8^ in patients with chronic hepatitis B (CHB) who achieve a functional cure,^5–7^ and in patients who control HBV after therapy discontinuation.^8–11^ In contrast, patients with CHB do not achieve cure display extremely low frequencies of HBV-specific T cells.^12–14^ This is a consequence of T cell exhaustion, whereby persistent viral replication, along with high antigen loads, result in functionally attenuated virus-specific T cells,^15–17^ and, ultimately, the most high-affinity cells being deleted. The remaining HBV-specific T cells are known to co-express multiple immune inhibitory receptors,^18–21^ exhibit metabolic deficiencies,^22–24^ and express an Eomes^hi^Tbet^lo^ transcriptional profile,^19,20,25,26^ which together culminate in T cell dysfunction. Given the evidence that T cell cytokines, such as interferon (IFN)-γ, can lead to the noncytolytic clearance of HBV,^27^ numerous clinical strategies have been proposed with the aim of restoring HBV-specific T cell functionality to achieve functional cure for CHB.

The PD-1/PD-L1 axis is a prime immunotherapeutic target against T cell exhaustion. Monoclonal α-PD-1 antibodies are approved for use in humans against many cancers, including melanoma, lung cancer, and renal cell carcinoma.^28–30^ The prospect of blocking the PD-1/PD-L1 axis to functionally restore exhausted HBV-specific T cells is supported by studies showing significantly more proliferation and higher IFN-γ secretion among HBV-specific T cells expanded in vitro.^13,31,32^ The first clinical use of α-PD-1 antibodies in patients with CHB led to a decline in HBsAg serum levels of ≥0.5 log_10_ among 3/12 patients, with a single patient achieving total HBsAg loss.^33^

While the potential is promising, previous studies only measured responsiveness to PD-1 blockade using *in vitro* expansion in HBeAg-inactive carriers (IC), where HBV-specific T cells are most detectable.^11,13,31,32,34,35^ No study has comprehensively investigated the effects of checkpoint blockade across the clinical phases of CHB *ex vivo* with a defined goal of identifying biomarkers that could predict response to therapy. Predicting responsiveness to checkpoint inhibitor therapies could stratify patients most likely to respond to treatment and minimize exposure to drugs that carry the potential for immune-related adverse events. Because only few patients with CHB have been treated with checkpoint inhibitors in Phase 1/2 clinical trials, we compared HBV-specific T cell functional restoration to an assay able to predict overall survival in cancer patients, the interferon response capacity (IRC).^36^ We demonstrate differential responses to PD-1 blockade by cytokine, clinical phase, and HBV antigen specificity. Furthermore, functional restoration by PD-1 blockade could be predicted by the IRC assay, providing a simplified flow cytometry assay that could predict CHB patient outcomes.

## METHODS

### Human subjects and whole blood processing

This study was approved by the Research Ethics Board at the University Health Network and conducted in accordance with both the Declarations of Helsinki and Istanbul. All patients with CHB were recruited at the Toronto Centre for Liver Disease and provided written informed consent. Peripheral blood mononuclear cells (PBMCs) were collected by density gradient centrifugation and cryopreserved in 90% Knockout Serum Replacement (Life Tech) + 10% DMSO (Sigma). Patients with CHB are classified by the American Association for the Study of Liver Disease (AASLD) guidelines; immunotolerant (IT) patients were HBeAg+, alanine transaminase (ALT) <1x upper limit of normal (ULN), viral load >10^6^ IU/mL, and high HBsAg levels. IA+ patients were HBeAg+, ALT >2xULN with viral load >20,000 IU/mL. IA− patients were HBeAg−, ALT >2xULN with viral load >2000 IU/mL. IC patients were HBeAg−, ALT <1xULN with HBV DNA <2000 IU/mL. Functionally cured (FC) patients were HBeAg−, HBsAg−, and HBV DNA negative. Patients on antiviral treatment (Tx) had viral load <10^3^ IU/mL and were HBeAg+/−. ALT ULN is defined as 35 U/L for males and 25 U/L for females. Supplemental Table S1, http://links.lww.com/HC9/A685 summarizes patient cohorts with CHB used for FluoroSpot assays, while Supplemental Table S2, http://links.lww.com/HC9/A685 summarizes patients with CHB used for liver biopsy stains.

### HBV overlapping peptide pools

Three hundred ten HBV genotype C, (Accession: AB112063) 15-mer peptides overlapping by 10 residues, purified to >70%, were purchased from GenScript. Peptides were dissolved in 100% DMSO to a concentration of 50 mg/mL. The 15-mer peptides were diluted in Aim-V medium and combined into 8 pools as described.^37^ All peptide pool stimulations were conducted at a final concentration of 5 µg/mL/peptide.

### Ex vivo total HBV-specific T cell stimulation

Cryopreserved PBMCs were thawed, resuspended in Aim-V medium (Life Tech) with 2% human serum (VWR International), and rested overnight at 37°C in 5% CO_2_ at 4×10^6^ PBMCs/mL. Following rest, PBMCs were counted and subjected to the 20:80 pulsing strategy as optimized.^37^

### Ex vivo HBV antigen-specific T cell stimulations

The 20:80 pulsing strategy was also used to measure *ex vivo* HBV antigen-specific responses. Five Eppendorf tubes each with 4.5×10^5^ PBMCs were centrifuged at 350*g* and resuspended in Aim-V with serum. Each tube was incubated with HBV overlapping peptide pool (OLP) pools corresponding to HBV Core, X, Env, or Pol (or DMSO equivalent) for 1 hour at 37°C in 5% CO_2_. Each of the pulsed PBMCs was centrifuged at 350*g* and each combined with 1.8×10^6^ unpulsed cells and resuspended in 1.125 mL Aim-V without serum. Each of the combined cells was plated at 4×10^5^ PBMCs/well (200 µL) in 5 wells.

### Ex vivo PD-1 blockade

HBV OLP pulsing was consistently performed for 1 hour at 37°C in 5% CO_2_. To assess the impact of PD-1 blockade on T cell responses, α-PD-1 blocking antibodies (BioLegend, clone: EH12.2H7) and respective isotype controls (BioLegend, clone: MG1-45) were added at a final concentration of 10 µg/mL concurrently with the HBV OLP pools during the 1 hour of peptide pulsing step. During the pulsing step, the unpulsed cells (1.8×10^6^ cells for every pulsed condition) were centrifuged at 350*g* and resuspended in 2×10^6^ cells/mL with Aim-V without serum and also incubated with α-PD-1 blocking antibodies or isotype controls (5 µg/mL). Following the 1 hour of pulsing, each of the pulsed 4.5×10^5^ PBMCs was centrifuged at 350*g* and resuspended at 2×10^6^ cells/mL with Aim-V without serum. Pulsed cells were respectively combined with 1.8×10^6^ unpulsed cells of the same blocking condition (ie, PD-1 blocked pulsed cells combined with PD-1 blocked unpulsed cells). The combined cells were likewise plated at 4×10^5^ PBMCs/well (200 µL) in 5 wells.

### 3-color FluoroSpot

FluoroSpot kits (Cellular Technologies Ltd.) detecting IFN-γ (green), IL-2 (yellow), and Granzyme B (red) secretion were coated as per the manufacturer’s protocol. All plates were incubated at 37°C for 24 hours before developing as per protocol. Granzyme B was uniformly negative and excluded from the analysis (not shown). Fluorescent readouts were counted using the C.T.L. ImmunoSpot S6 Analyzer (Cellular Technology Limited).

### Ex vivo detection of HBV-specific T cells by means of flow cytometry

For the *ex vivo* total HBV-specific T cell stimulation, similar to the method described above, PBMCs of patients with CHB were resuspended in Aim-V medium containing 2% human serum and 20% were loaded with the combined HBV OLP pools, mixed and cultured for 24 hours. In the last 4 hours of culture, Brefeldin A (1 ug/mL, Sigma) and Monensin (2 µM, Biolegend) were added to the media. Following incubation, cells were stained with LIVE/DEAD Fixable Near-IR Dead Cell Stain reagent for 10 minutes at room temperature, washed with FACS buffer (PBS supplemented with 2% FBS), and surface labeled with an antibody cocktail containing CD3-R718 (BD, clone SP34-2), CD4-APC-H7 (BD, clone RPA-T4), CD8-BUV395 (BD, clone RPA-T8) for 30 minutes at 4°C in the dark. After washing with FACS buffer, cells were fixed using Cytofix/Cytoperm (BD Biosciences) for 20 minutes at 4°C, washed with Perm-wash buffer (PBS containing 10% FBS and 0.1% saponin) and stained with IFNg-APC (BioLegend, clone B27), and IL-2-PE (BioLegend, clone MQ1-17H12) in Perm-wash buffer for 30 minutes at 4°C. Cells were fixed in 1% paraformaldehyde until acquisition using a BD Symphony A3 flow cytometer and analyzed with FlowJo V10.

### Immunohistochemistry in CHB liver biopsies

Formalin-fixed biopsies were deparaffinized, rehydrated, and subjected to antigen retrieval using citrate pH 6.0 (for HbcAg), Tris-EDTA pH 9.0 (for CD4 and CD8), pepsin (for CD68), or commercial CC1 buffer Ventana (for PD-1 and PD-L1). Sections were then incubated with antibodies to the following: HBsAg (clone A10F1, 1:100, Cell Marque, Rocklin, CA), HbcAg (1:800, Abcam, Cambridge, UK), CD4 (clone 4B12, 1:200, Leica, Buffalo Grove, IL), CD8 (clone 4B11, 1:1000, Leica, Buffalo Grove, IL), CD68 (clone PG-M1, 1:300, Dako, Santa Clara, CA), PD-1 (clone NAT105, 1:200, Abcam, Cambridge, UK), PD-L1 (clone 28-8, 1:200, Abcam, Cambridge, UK). Expression of the markers was quantified using the Visiopharm software, by dividing the number of strongly positive cells by the total stained area. PD-L1 staining on hepatocytes was scored on a semi-quantitative scale: 1+ 25% positive, 2+ as intermediate, and 3+ diffuse staining.

### Mass cytometry immunostaining and analysis

Cells were stained according to the protocol described previously^36^. Briefly, following PBS washes, cells were incubated for 15 minutes at 37°C with 1 μM Cell-ID Intercalator-Rh (Standard BioTools) prepared in RPMI, to label dead cells for exclusion. Cells were fixed for 10 minutes with Foxp3 Fixation/Permeabilization buffer (Thermo Fisher Scientific), and each sample was labeled with a unique barcode using the Cell-ID 20-Plex Pd Barcoding Kit (Standard BioTools). After barcoding, samples were pooled into a single tube and incubated for 10 minutes with Fc-receptor blocking reagent. Surface antibodies were diluted in cell staining buffer (PBS + 2% FBS), and cells were incubated with the surface antibody mix for 30 minutes at 4°C. After washing, cells were then incubated with intracellular staining mix (prepared in permeabilization wash buffer) for 30 minutes at 4°C, followed by a 1-hour incubation with 125 nM of Cell-ID Intercalator-Ir (Standard BioTools) prepared in PBS with 0.3% saponin + 1.6% paraformaldehyde. Samples were stored in PBS + 1.6% paraformaldehyde until acquisition. Samples were acquired on a Helios instrument at the Princess Margaret Cancer Center.

Using FlowJo 10 (v10.8.1), the normalized FCS file was de-barcoded to generate a separate FCS file for each sample. A sequential gating strategy was used to select live CD45^+^ events by first gating total cells, filtering out doublets, beads, and dead cells followed by identifying CD45^+^ cells. The single-cell signal intensities of CD45^+^ events were exported as CSV files and analyzed in R (v 4.2.1). First, marker expression values were arcsinh transformed using a custom cofactor for each marker. Then fast-PhenoGraph (v.0.0.6) was run using 2 different sets of markers, one to obtain clusters of cells based on immune phenotype (using lineage-defining markers for clustering) and another to identify cells expressing similar patterns of interferon-stimulated proteins (ISPs) (modules). Phenotypically similar clusters were grouped together based on their biological subset. Finally, UMAP was run using lineage-defining markers to facilitate visualization of the entire immune compartment in 2 dimensions.

### IRC assay

PBMCs were resuspended in AIM-V media (no FBS/AB serum) and seeded in 24-well ultra-low attachment plates (Corning) at a density of 1 × 10^6^ cells/mL and incubated for 1 hour at 37°C to recover. Next, cells were left unstimulated in AIM-V medium or stimulated with 1000 U/mL of recombinant human IFN-β for 16 hours. Finally, Brefeldin A (1 ug/mL, Sigma) and Monensin (2 µM, Biolegend) were added to the culture media and incubated for an additional 2 hours.

Following incubation, cells were labeled with Zombie Aqua Viability Dye (BioLegend) for 20 minutes at 4°C in the dark, washed with FACS buffer, and stained with a surface antibody cocktail containing CD3-PE/Cy7 (BioLegend, clone UCTH1, 1:400), CD4-APC (BioLegend, clone RPA-T4, 1:100), CD8-AF700 (BioLegend, clone RPA-T8), and CD11c-PE/Dazzle594 (BioLegend, clone Bu15), for 15 minutes at 4°C in the dark. For intracellular staining, the Foxp3 Staining Kit (Thermo Fisher Scientific) was used according to the manufacturer’s instructions. Cells were fixed with Foxp3 Fixation/Permeabilization buffer for 30 minutes at room temperature and then washed with 1× permeabilization buffer. Cells were stained for 45 minutes at room temperature with ISG15-PE (R&D Systems, clone 851701) in 1× permeabilization buffer. Data were acquired on the Symphony A3 flow cytometer (BD, Biosciences) and analyzed using FlowJo v.10.

### Statistical analysis

All statistical analyses were conducted in GraphPad Prism (version 8.4.3). Details of each statistical test are included in each figure legend.

## RESULTS

### Differential production of IFN-γ and IL-2 across the clinical stages of CHB

**FIGURE 1:**
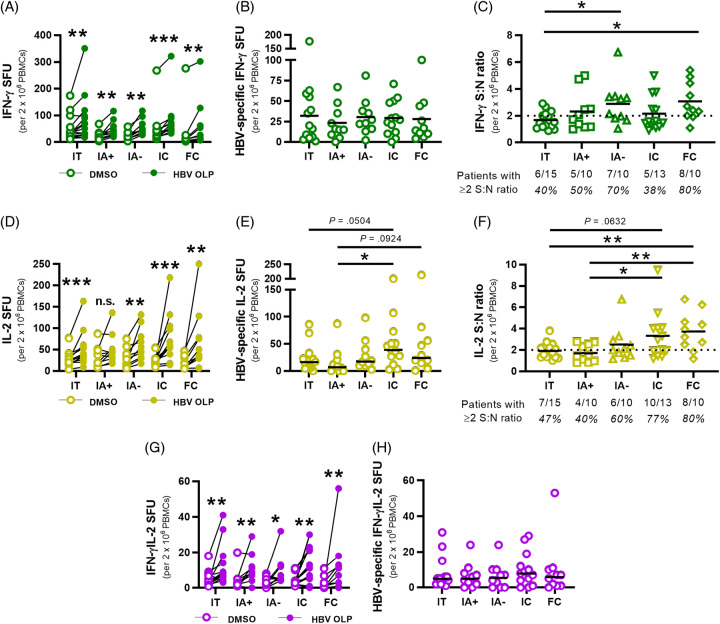
*Ex vivo* detection of HBV-specific T cells in patient cohorts with chronic hepatitis B by means of multianalyte FluoroSpot assays. (A) IFN-γ+ SFU for HBV OLP (filled circles) and DMSO (empty circles) conditions were linked per patient in each cohort. (B) IFN-γ+ HBV-specific SFU for each patient were calculated by subtracting DMSO SFU from HBV OLP SFU. (C) IFN-γ+ signal:noise (S:N) ratios were calculated for each patient by dividing HBV OLP SFU by DMSO SFU. Ratios ≥2 were considered positive responses. Fractions beneath each cohort denote the number of patients with detectable responses. Black lines indicate sample means. (D) IL-2 + SFU for HBV OLP and DMSO conditions linked per patient in each cohort. (E) IL-2+ HBV-specific SFU for each patient from subtracting DMSO SFU from HBV OLP SFU. (F) IL-2+ S:N ratios for each patient by dividing HBV OLP SFU by DMSO SFU in each patient. (G) Multifunctional IFN-γ+IL-2+ SFU for HBV OLP and DMSO were linked per patient. (H) HBV-specific IFN-γ+IL-2+ SFU for each patient from subtracting DMSO SFU from HBV OLP SFU. Wilcoxon tests were conducted to compare SFU between HBV OLP and DMSO conditions (A, D, G). Mann-Whitney tests were used to compare HBV-specific SFU and S:N ratios between patient cohorts (B, C, E, F, H) (*p<0.05, **p<0.01, ***p<0.001). Abbreviations: FC, functionally cured; IA, immune-active; IC, inactive carrier; IFN-γ, interferon; IT, immunotolerant; OLP, overlapping peptide pool; PBMC, peripheral blood mononuclear cells; S:N ratio, signal-to-noise ratio; SFU, spot-forming unit.

Having optimized the FluoroSpot assay to effectively measure HBV-specific T cell responses in patients with CHB *ex vivo*,^37^ our initial aim was to compare functional HBV-specific T cell responses across the adult phases of chronic HBV infection. PBMCs were isolated from immune tolerant (IT; n=15), HBeAg+ immune-active (IA+; n=10), HbeAg− immune-active (IΑ−; n=10), ICs (n=13), and FC (n=10) patients categorized by the American Association for the Study of Liver Disease guidelines (Supplemental Table S1, http://links.lww.com/HC9/A685). Total T cell responses to HBV were evaluated by stimulating with HBV OLP and assessed for IFN-γ and IL-2 secretion. DMSO alone served as a negative control. We found significant increases in IFN-γ+ cells in all patient cohorts on HBV OLP stimulation over DMSO alone (Figure 1A). HBV-specific (background subtracted) spot-forming units (SFU) revealed no significant differences in IFN-γ+ HBV-specific T cell counts across phases (Figure 1B). We calculated signal-to-noise (S:N) ratios to account for the different magnitudes of responses between patients, and used a positive cutoff of S:N ≥ 2 to further compare IFN-γ+ responses across clinical stages (Figure 1C). From the S:N ratios, IT patients displayed the weakest IFN-γ+ response compared to other cohorts. IΑ- and FC patients had significantly stronger IFN-γ+ T cell responses than IT patients (Figure 1C, *p*=0.023, 0.0096). The percent of patients with a defined positive IFN-γ response based on S:N ratios are indicated beneath each phase (Figure 1C). The FC cohort reported the strongest IFN-γ+ ratios and the greatest number of detectable responses 8/10 (80%). Both IT and IC cohorts reported the least responders with 6/15 (40%) IT and only 5/13 (38%) IC patients based on IFN-γ+ S:N ratios.

HBV-specific IL-2 responses were detectable in all cohorts except IA+ patients on HBV OLP stimulation (Figure 1D). In contrast to HBV-specific IFN-γ+ responses that did not differ significantly between phases (Figure 1B), HBV-specific (background subtracted) IL-2+ SFU were significantly higher in IC compared to IA+ patients (*p*=0.0185) and approached significance between IC and IT patients (Figure 1E). S:N ratios of IL-2+ responses showed a general trend of higher IL-2 ratios among cohorts exhibiting greater viral control, with both IC and FC patients displaying significantly higher S:N ratios than IA+ patients (Figure 1F, *p*=0.0303, 0.0068), and FC against IT patients (*p*=0.0064). IL-2+ S:N ratios were observed in 10/13 (77%) IC and 8/10 (80%) FC patients. In contrast, only 7/15 (47%) IT and 4/10 (40%) IA+ patients had detectable IL-2+ responses, representing the 2 cohorts with the lowest S:N ratios and the least number of detectable responses.

Multifunctional IFN-γ+IL-2+ cells could be detected in all cohorts on HBV OLP stimulation (Figure 1G). When we compared the HBV-specific (background subtracted) multifunctional responses, we did not observe significant differences between the cohorts (Figure 1H). Given the minimal background for multifunctional SFU, S:N ratios were not used.

Our results show *ex vivo* detection of both IFN-γ+ and IL-2+ HBV-specific T cells across all CHB cohorts. Responses for IFN-γ showed no differences in absolute HBV-specific SFU, but S:N ratios were significantly elevated in patients who achieved functional cure and in patients with HBeAg-negative hepatitis. IC patients displayed significantly higher IL-2+ cells and both IC and FC patients had significantly higher IL-2 S:N ratios than all other CHB cohorts, suggesting IL-2 production was associated with robust viral control.

### Ex vivo PD-1 blockade differentially impacts cytokine responses across chronic HBV phases

**FIGURE 2:**
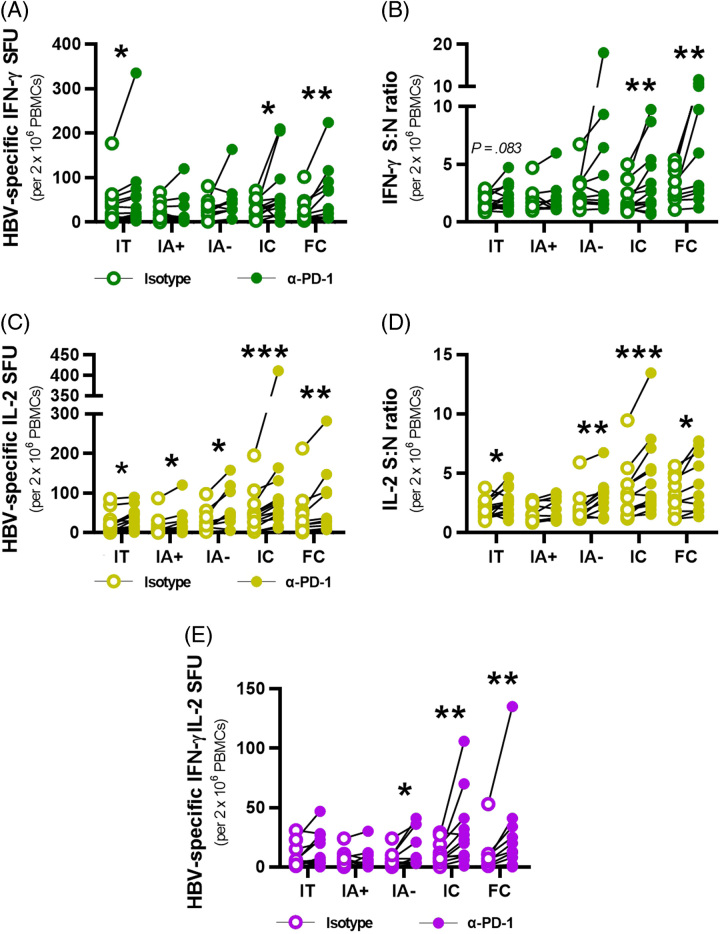
*Ex vivo* α-PD-1 blocking leads to functional restoration of HBV-specific T cells among patients with chronic hepatitis B. (A) HBV-specific IFN-γ+ SFU for each patient were calculated by subtracting DMSO SFU from HBV OLP SFU for isotype- (empty circles) and α-PD-1 blocked (filled circles) conditions. (B) IFN-γ+ S:N ratios were calculated for each patient by dividing HBV OLP SFU by DMSO SFU for each blocking condition. (C) HBV-specific IL-2+ SFU for each patient from subtracting DMSO SFU from HBV OLP SFU per blocking condition. (D) IL-2+ S:N ratios for each patient by dividing HBV OLP SFU by DMSO SFU per blocking condition. (E) Multifunctional IFN-γ+IL-2+ HBV-specific SFU for each patient following background subtraction for each blocking condition. Wilcoxon tests were conducted to compare HBV-specific SFU and S:N ratios between isotype- and α-PD-1 blocked conditions in each cohort (A–E) (*p<0.05, **p<0.01, ***p<0.001). Abbreviations: FC, functionally cured; IA, immune-active; IC, inactive carrier; IFN-γ, interferon; IT, immunotolerant; PBMC, peripheral blood mononuclear cells; S:N ratio, signal-to-noise ratio; SFU, spot-forming unit; PD-1, programmed cell death ligand protein 1.

Having defined T cell responsiveness across different CHB phases, we determined if these functional responses could be enhanced through PD-1 blockade *ex vivo*. CHB PBMCs were stimulated with OLPs in the presence of α-PD-1 blocking antibodies or an isotype control antibody. On PD-1 blockade, IFN-γ+ HBV-specific T cells were significantly higher among IT, IC, and FC patients in response to HBV antigen stimulation (Figure 2A, *p*=0.0157, *p*=0.0181, 0.0039). IFN-γ S:N ratios were significantly increased on PD-1 blockade among IC and FC patients (Figure 2B, *p*=0.0081, 0.0020).

We then assessed IL-2 responses to PD-1 blockade. In contrast to IFN-γ, PD-1 blockade significantly increased IL-2+ HBV-specific SFU in all cohorts of patients with CHB, demonstrating that IL-2 functionality is more readily restored than IFN-γ (Figure 2C). PD-1 blockade significantly increased S:N ratios in all cohorts except for IA+ patients (Figure 2D). Multifunctional responses were significantly improved on PD-1 blockade in IΑ, IC, and FC patients (*p*=0.0156, 0.0039, 0.0039), confirming the capacity to also restore polyfunctional HBV-specific T cell responses (Figure 2E). Neither IFN-γ nor IL-2 DMSO SFU (background) were found to significantly differ on PD-1 blockade (Supplemental Figure S1, http://links.lww.com/HC9/A685). These data demonstrate that ex vivo PD-1 blockade leads to enhanced cytokine responses with the most significant elevation observed in patients with the strongest viral control: IC and FC patients.

### HBeAg+ immune-active patients demonstrate the least capacity to respond to ex vivo immune checkpoint blockade

**FIGURE 3:**
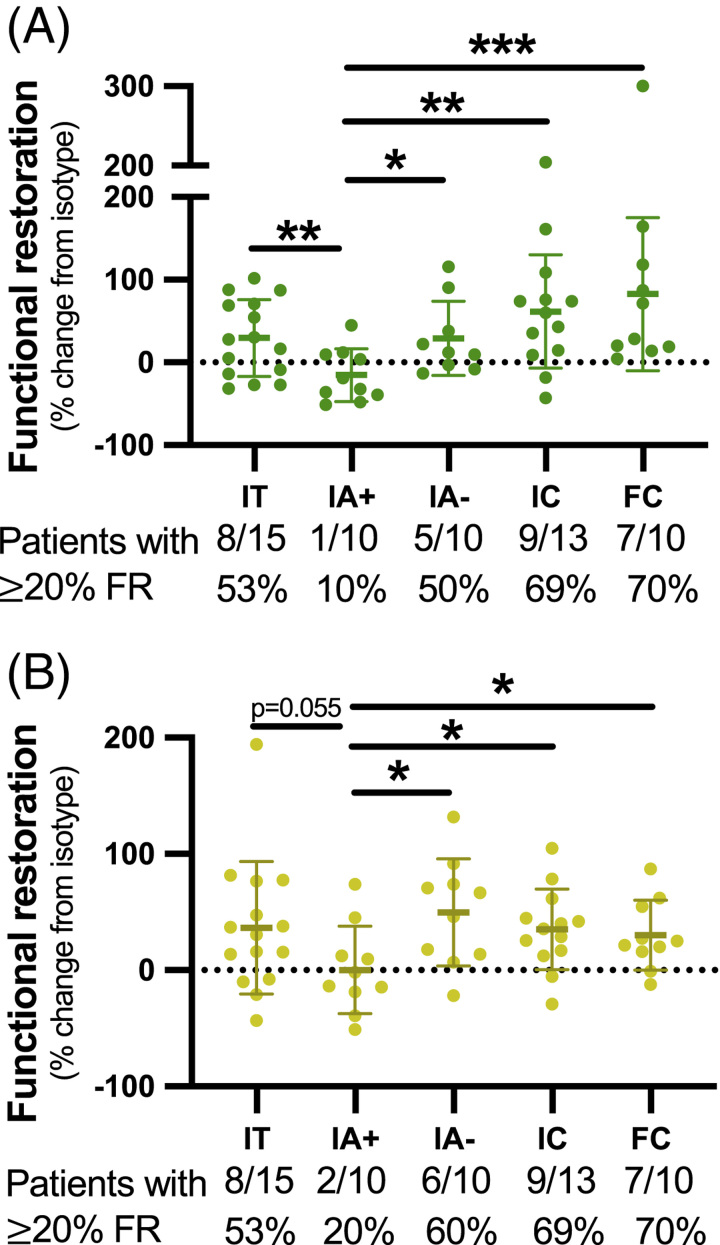
Functional restoration from *ex vivo* PD-1 blockade across patient cohorts with chronic hepatitis B. Functional restoration was calculated and tabulated per patient for (A) IFN-γ and (B) IL-2 responses. The dotted line represents 0%, while full black lines indicate means with SDs. Fractions beneath each cohort indicate the number of patients having a functional restoration percentage of ≥20% and considered treatment-responsive to *ex vivo* PD-1 blockade. Mann-Whitney tests were used to compare functional restoration between patient cohorts (A, B) (* *p*<0.05, ** *p*<0.01, *** *p*<0.001). Abbreviations: FC, functionally cured; FR, functional restoration; IA, immune-active; IC, inactive carrier; IFN-γ, interferon; IT, immunotolerant PD-1, programmed cell death protein 1.

As previously shown,^37^ the use of S:N ratios accounts for inter-patient variability and could allow for stratification of chronic patients for checkpoint inhibitor therapy by their *ex vivo* HBV-specific T cell responses. To calculate the efficacy of PD-1 blockade, we calculated the differences in S:N ratios between isotype and PD-1 blocked samples (Figure 3). IC and FC patients exhibited the greatest average increase in IFN-γ+ functional restoration (Figure 3A). IA+ patients were virtually nonresponsive to PD-1 blockade with only 1/10 IA+ patients recording ≥20% increase in IFN-γ response on checkpoint blockade. IC and FC patient groups had the highest proportions of patients achieving ≥20% gain in IFN-γ production in response to ex vivo PD-1 blockade (69% and 70%, respectively; Figure 3A). Functional restoration of IL-2+ responses was observed across all cohorts apart from IA+ patients. Only 2/10 IA+ patients displayed a ≥20% increase in IL-2 signal:noise (Figure 3B). Again, IC and FC cohorts had the highest percentage of patients with increased IL-2 production (69% and 70%, respectively; Figure 3B). Overall, these data show that patients in the IA+ cohort respond poorly to checkpoint blockade, whereas all other cohorts displayed a measure of benefit to PD-1 blockade.

### PD-1 and PD-L1 expression in liver tissue by CHB clinical phase

**FIGURE 4:**
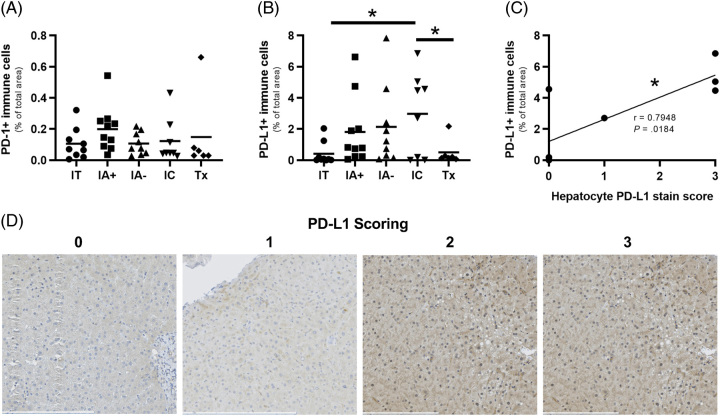
Liver biopsy stains for PD-1/PD-L1 across patient cohorts with chronic hepatitis B. (A) Area of PD-1+ expression among intrahepatic immune cells across chronic hepatitis B cohorts. (B) Area of PD-L1+ expression among intrahepatic immune cells across chronic hepatitis B cohorts. (C) Correlation between PD-L1+ area among intrahepatic immune cells and PD-L1 scoring on hepatocytes among IC patients. (D) Representative PD-L1 staining on hepatocytes to demonstrate weak (left) and diffuse (right) stains, black scale bars represent specified lengths. Unpaired parametric *t* tests were conducted to compare marker expression between cohorts (A, B). Pearson correlation coefficient and *p-*values are listed (C). (*p<0.05). Abbreviations: IA, immune-active; IC, inactive carrier; IT, immunotolerant; PD-1, programmed cell death protein 1; PD-L1, programmed cell death ligand 1; Tx, treatment.

In addition to the importance of PD-1 on peripheral T cells, PD-1 and PD-L1 expression in the liver may differ by CHB phase and impact response to PD-1 blockade. Tissue from formalin-fixed paraffin-embedded liver biopsy samples from IT (n=9), IA+ (n=10), IA− (n=9), and IC (n=8) patients, as well as those on antiviral therapy (Tx, n=6), were stained for PD-1 and PD-L1 (Supplemental Table S2, http://links.lww.com/HC9/A685). PD-1 staining did not differ in liver biopsies by clinical phase (Figure 4A). The proportion of PD-L1+ immune cells was greatest in the IC group, which was significantly higher than the IT (*p*=0.0137) and treated cohorts (*p*=0.0494) (Figure 4B). PD-L1 staining in hepatocytes was strongest in the IC and IT cohorts. Diffuse PD-L1 hepatocyte staining (3+) was seen in 3/8 IC (37.5%) and 1/9 IT (11.1%) patients with the weakest hepatocyte staining seen among the IA+ and treated cohorts. We observed a strong correlation between immune cell and hepatocyte PD-L1 staining in the IC patients (Figure 4C, *r*=0.7948, *p*=0.0184) but not in other groups. Representative stains of weak and diffuse PD-L1 staining on hepatocytes are demonstrated (Figure 4D). PD-1 and PD-L1 staining did not correlate with HBsAg or HBcAg staining. These data show that IC patients, who were most responsive to PD-1 blockade, express the highest level of PD-L1 in the liver.

### Ex vivo HBV-specific T cell hierarchies are distinct between chronic HBV phases

Understanding the antigen hierarchy in chronic HBV infection can lend further insight into how HBV-specific T cell immunity is shaped over the course of infection and determine which T cell specificities are associated with functional restoration. We characterized the *ex vivo* hierarchy of HBV-specific T cells targeting different viral antigens among IT and IC patients. These 2 cohorts represent the extreme ends of minimal (IT) and robust (IC) viral control. We used patients with a confirmed IFN-γ or IL-2 response to HBV OLP (Figure 1) and stimulated PBMCs from IT (n=9) and IC patients (n=10) with HBV OLP pools by antigen [Core, X, Envelope (Env), Polymerase (Pol)].

**FIGURE 5:**
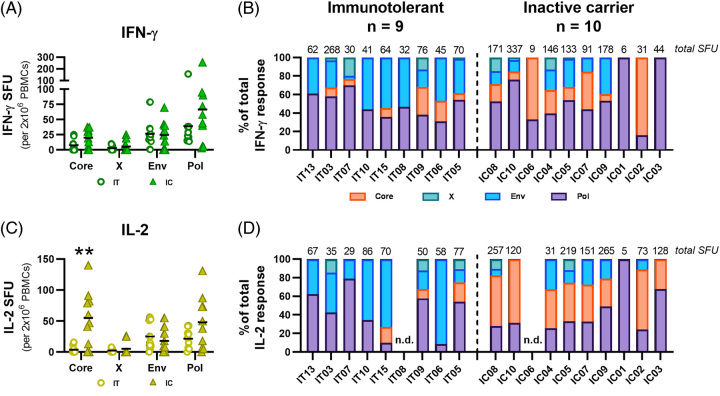
*Ex vivo* HBV antigen hierarchies are distinct between chronic hepatitis B phase. (A) IFN-γ+ SFU for IT (circles) and IC patients (triangles) were calculated by subtracting DMSO SFU from each HBV overlapping peptide pool antigen SFU. Black lines indicate sample means. (B) Total IFN-γ SFU are listed above each patient and the proportions of IFN-γ responses were calculated for each HBV antigen. (C) IL-2+ SFU for IT and IC patients were calculated by subtracting DMSO SFU from each HBV overlapping peptide pool antigen SFU. (D) Total IL-2 SFU are listed for each patient and the proportions of IL-2 responses were calculated for each HBV antigen. Mann-Whitney tests were used to compare antigen-specific SFU between IT and IC patients (A, C) (**p<0.01). Abbreviations: IC, inactive carrier; IFN-γ, interferon; IT, immunotolerant; n.d., not detected; SFU, spot-forming unit.

No HBV antigen exhibited significantly different IFN-γ+ responses between IT and IC patients. IT patients, on average, had less Core- and Pol-specific IFN-γ+ cells than IC patients (Figure 5A). Patients were individually assessed to determine their HBV antigen hierarchies. The total magnitude of each HBV antigen-specific spot count is listed for each patient at the top of the graphs (Figure 5B, D). HBV Pol accounted for the majority of IFN-γ+ responses in 5/9 (56%) IT and 8/10 (80%) IC patients (Figure 5B). Despite detecting no differences in IFN-γ+ Env-specific responses between cohorts (Figure 5A), Env-specific responses comprised a greater proportion of the overall response in IT patients due to reduced Core- and Pol-specific responses (Figure 5A, B). We also analyzed the impact of age on Env-specific T cell frequency (n=19) but did not find a significant correlation with patient age (Supplemental Figure S2, http://links.lww.com/HC9/A685). However, it should be noted that our IT cohort was primarily composed of adult patients, with limited data in patients under 30 years old. HBx-specific T cells were weak and did not differ between cohorts or by cytokine (Figure 5A, B).

Among IL-2 responses, Core-specific responses were significantly higher among IC patients compared to IT patients (Figure 5C, *p*=0.0049). IL-2+ Pol-specific cells tended to be higher among IC than IT patients; however, this was not statistically significant. Analysis of IL-2+ antigen hierarchies revealed a robust Core-dominant IL-2 response in 6/10 (60%) IC patients that was not observed among IT patients (Figure 5D). IL-2+ Env-specific dominance was observed among 4/9 (44%) IT patients, but similar to IFN-γ, Env-specific responses made up a larger proportion of *ex vivo* responses due to reduced Core- and Pol-specific responses in IT patients. IL-2+ HBV Pol-specific responses were detected in majority of both IT and IC patients.

The ex vivo antigen-specific analyses revealed distinct differences among HBV-specific T cells between IT and IC patients. While Env- and Pol-specific responses were widely observed to be equal between the 2 cohorts, IC patients displayed a prevalent response against Core that was nearly absent among IT patients.

### Differential T cell restoration between HBV antigens, clinical phase, and cytokine response

**FIGURE 6:**
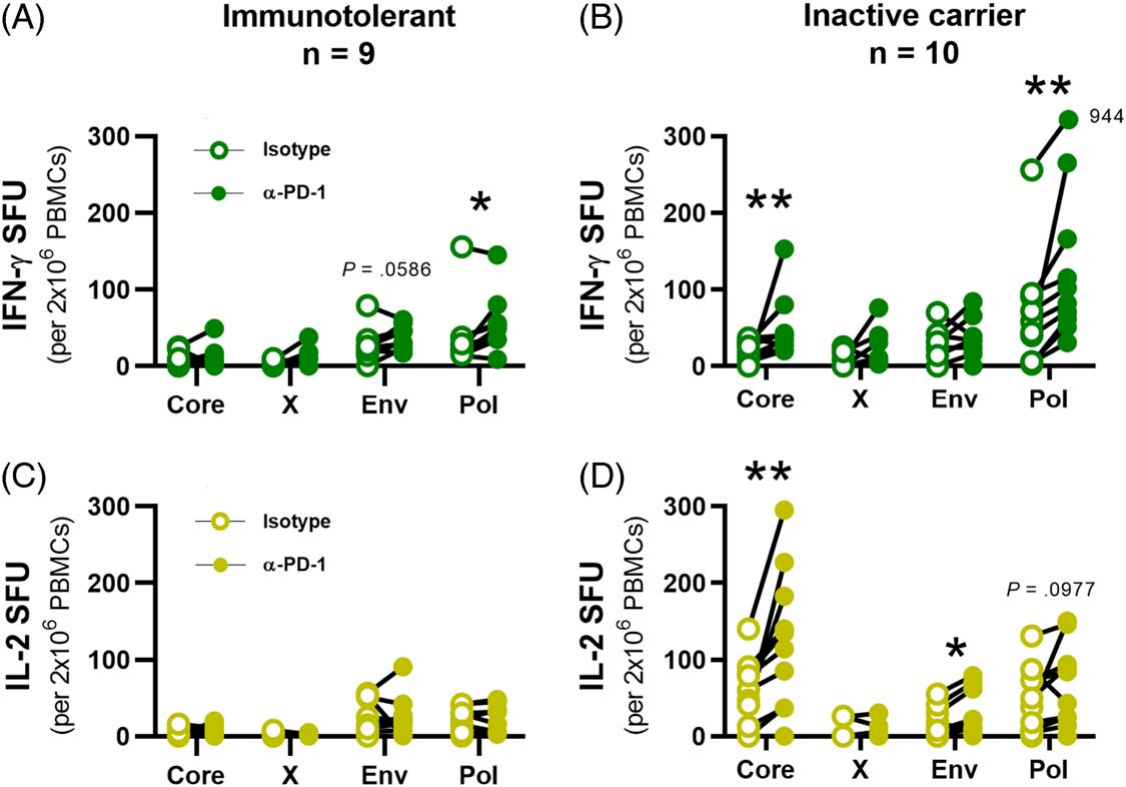
Differential functional restoration of HBV antigen-specific T cell responses by clinical phase and cytokine response. Ag-specific IFN-γ+ SFU for (A) IT and (B) IC patients were each calculated for isotype- (empty circles) and α-PD-1 blocked (filled circles) conditions. Ag-specific IL-2+ SFU for (C) immunotolerant and (D) inactive carrier patients between isotype- and α-PD-1 blocked conditions. Wilcoxon tests were conducted to compare Ag-specific SFU between isotype- and α-PD-1 blocked conditions (*p<0.05, **p<0.01). Abbreviations: IFN-γ, interferon; PBMCs, peripheral blood mononuclear cells; PD-1, programmed cell death protein 1; SFU, spot-forming unit.

The above data demonstrate that the stage of CHB impacts antigen-specific T cell hierarchy. T cells specific to different epitopes display different profiles of exhaustion markers,^19–21^ suggesting that virus-specific T cells within the same patient will respond differently to PD-1 blockade depending on the stage of infection.^19–21^ Ag-specific stimulations were carried out in the presence of α-PD-1 blocking antibodies or isotype controls. Significant restoration of IFN-γ+ production in IT patients was only observed in Pol-specific T cells (Figure 6A, *p*=0.0273). Restoration of Env-specific responses on α-PD-1 blocking was detected among IT patients but did not reach significance (*p*=0.0586). IC patients demonstrated significantly higher responses for both Pol-specific and Core-specific IFN-γ+ cells on PD-1 blockade (Figure 6B, *p*=0.0020, 0.0098). No IL-2+ responses were significantly restored among IT patients (Figure 6C), whereas IC patients showed significant increases in Core-specific and Env-specific responses (Figure 6D, *p*=0.0039, 0.0234). These data demonstrate that the most robust T cell responses in IC patients (Core and Pol) were also effectively enhanced on PD-1 blockade, suggesting that α-PD1 preferentially boosts function in T cells that retain some level of activity.

### The type I IFN response capacity in CD4 T cells significantly correlates with functional restoration on PD-1 blockade

Up to this point, responses were measured using Fluorospot assays, without discriminating between CD4 and CD8 T cells. However, the association of IL-2 with better HBV control, and previous reports demonstrating the importance of HBV-specific CD4 T cells in patients who achieve functional cure naturally or after stopping nucleoside analog therapy,^7,9^ prompted us to investigate IL-2 production by intracellular cytokine staining. HBV-specific CD4 T cells were the dominant producers of IL-2 in patients with CHB (Supplemental Figure S3, http://links.lww.com/HC9/A685). Therefore, CD4 T cell responsiveness to α-PD1 therapies may represent a key axis for functional cure in patients with CHB.

**FIGURE 7:**
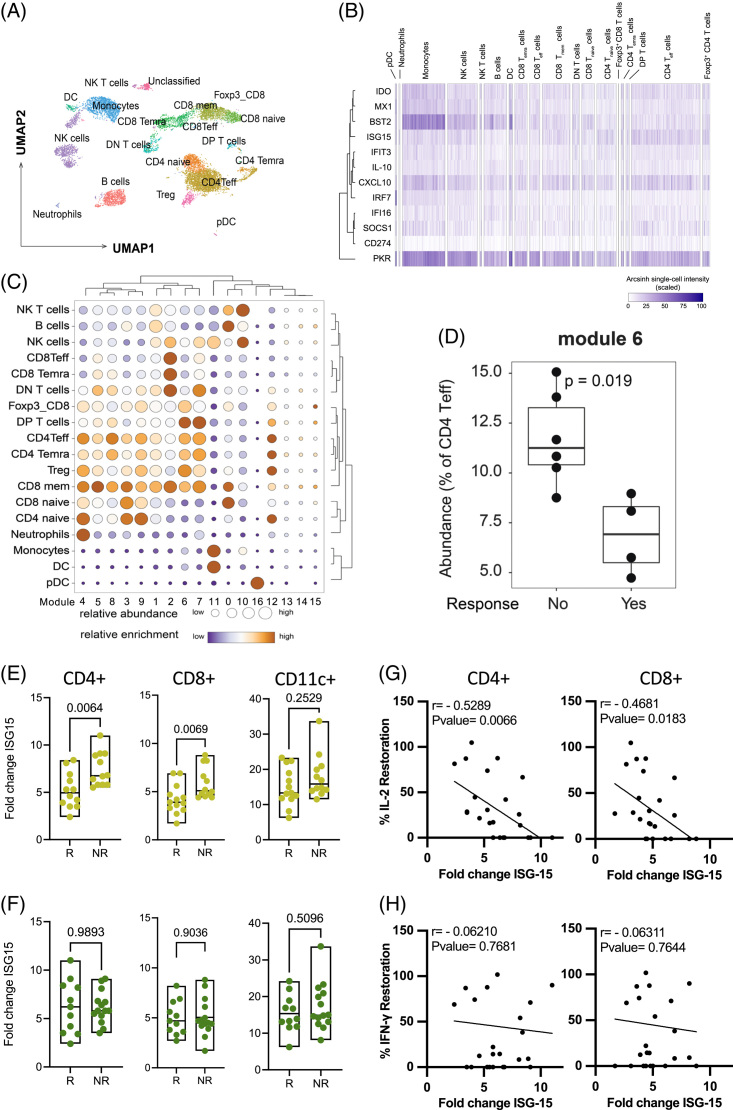
IFN response capacity correlates with IL-2 restoration after PD-1 blockade. (A) UMAP projection of immune cell populations identified in the cytof staining panel. (B) Single-cell heatmap showing the expression pattern of 12 interferon-stimulated proteins across the immune populations defined in (A). The transformed expression of each protein is scaled as a percentage of maximum expression. (C) Fast-Phenograph was used to define distinct modules of interferon-stimulated proteins. The relative abundance of each module within a given cell subset (size of dots) and the enrichment of the cell subset within a module (color of dots) are shown for the 16 modules. (D) Frequency of CD4 Teff cells positive for interferon-stimulated protein module 6 between anti-PD1, IL-2+IFN-γ+ responder (n=6) and nonresponder (n=4) patients. Boxes show the median, upper, and lower quartile and whiskers extend to 1.5X the interquartile range. Patients were stratified to responder/nonresponders to anti-PD-1 based on their (E) IL-2 (yellow) (R n=13; NR n=12 ) or (F) IFN-γ (green) (R n=11 ; NR n=14 ) HBV-specific T cell FluoroSpot results. Fold change in ISG15 expression in CD4 T cells, CD8 T cells, and myeloid cells was calculated by dividing the mean fluorescent intensity of IFN-β-stimulated cells by that of the unstimulated sample. Nonparametric Mann-Whitney *t* test was used for the statistical analysis. (G) Correlation between the percent increase in IL-2 production after PD-1 blockade and fold change in ISG15 expression. (H) Correlation between the percent increase in IFN-γ production after PD-1 blockade and fold change in ISG15 expression. Abbreviations: BST2, bone marrow stromal cell antigen 2; CD274, cluster of differentiation 274; CXCL-10, C-X-C motif chemokine 10; DC, dendritic cell; DN, double negative; DP, double positive; IDO, Indoleamine 2,3-dioxygenase; IFI16, interferon gamma inducible protein 16; IFIT3, interferon induced protein with tetratricopeptide repeats 3; IFN-γ, interferon; IRF7, interferon regulatory factor 7; ISG15, interferon stimulated gene 15; MX1, Myxovirus resistance protein 1; NK, natural killer cell; NR, nonresponder; PD-1, programmed cell death protein 1; pDC, plasmacytoid dendritic cell; PKR, protein kinase R; R, responder SOCS1, suppressor of cytokine signaling 1; Treg, regulatory CD4 T cell; UMAP, uniform manifold approximation and projection.

To support our observation that PD-1-responsive, IL-2-producing CD4 T cells may predict outcomes in patients with CHB, we adapted an approach that predicts overall survival in patients with cancer receiving α-PD1 therapy.^36^ The IFN-I response capacity (IRC) assay measures the induction of ISPs in total effector CD4 T cells following overnight exposure to IFN-β. As a pilot, we compared ISP expression in patients most responsive to PD-1 blockade (both IL-2 and IFN-γ increase in Fluorospot) to those least responsive (no changes). Mass cytometry (CyTOF) was performed on PBMC from 10 patients with CHB using a panel of 38 antibodies to measure the expression of 12 ISPs and 28 lineage- and state-defining proteins (Figure 7A). Single-cell ISP expression patterns varied across immune cell subsets with monocytes bearing the highest levels of ISPs (Figure 7B). When ISPs were used to cluster cells, we identified 17 distinct patterns of ISP expression (modules) that were differentially enriched across immune cells (Supplemental Figure S4A, http://links.lww.com/HC9/A685, Figure 7C). Specifically, module 6 was abundant across multiple T cell subsets and showed the highest expression of SOCS1, IDO, and CXCL10 (Figure 7C, Supplemental Figure S4A, http://links.lww.com/HC9/A685). We compared the level of ISP expression in module 6, and frequency of cells positive for ISPs, to restoration associated with PD-1 blockade. The level of ISPs (similar to mean fluorescence intensity) expressed in CD4 T cells was not different for any module between PD-1 responders and nonresponders (NRs) (Supplemental Figure S4B, http://links.lww.com/HC9/A685). However, the percentage of CD4 T cells expressing ISPs in module 6 in NRs was significantly higher compared to patients who responded to PD-1 blockade in the *ex vivo* fluorospot assay (Figure 7D). These data suggest the IRC predicts improved IL-2 production in response to PD-1 blockade and, therefore, may correlate with response to PD-1 therapy.

In patients with metastatic melanoma, or non-small cell lung cancer, the *in vitro* responsiveness of PBMC-derived CD4 T cells to IFN-β was more effective at predicting subsequent response to α-PD1 therapy than was the state of the CD4 T cells directly *ex vivo*.^36^ In particular, CD4 T effector cells from patients who responded to α-PD1 counter-intuitively exhibited lower responsiveness to IFN-β. Patients with CHB were stratified into 2 groups based on IL-2 production after PD-1 blockade (1) responder (R): increased IL-2 spots after PD-1 blockade and (2) NRs: no change in IL-2 spots after PD-1 blockade. We stimulated total PBMC overnight with IFN-β and measured ISG15 (an ISP) expression in CD4 T cells, CD8 T cells, and monocytes (Supplemental Figure S4C, http://links.lww.com/HC9/A685). Consistent with cancer patient data, responders (increased IL-2) exhibited a significantly lower IRC in CD4 and CD8 T cells compared to nonresponding patients (Figure 7E;^36^). When patients were stratified based on IFN-γ responsiveness, we did not observe any significant differences (Figure 7F). CD11c^+^ myeloid cells were not significantly different for any comparison (Figure 7E, F). Furthermore, the IRC fold change inversely correlated with IL-2 restoration in both CD4 and CD8 T cells (Figure 7G) while IFN-γ restoration did not (Figure 7H). The significant correlation between IL-2 restoration in the fluorospot assay and the IRC supports our hypothesis that response to α-PD-1 therapies may be predictable in patients with CHB.

## DISCUSSION

Novel drugs targeting different aspects of the immune system are being developed for CHB therapy but suffer from a lack of immunological biomarkers that can predict responses to treatment or durability of HBsAg loss.^38,39^ These biomarkers may be particularly important for immune checkpoint inhibitors, where the risk of immune-related adverse events is a significant concern relative to the safety of nucleoside analog therapy. To begin addressing this gap, we used our optimized fluorospot assay to measure T cell responsiveness to PD-1 blockade across the different phases of chronic HBV infection.^40^ Our results indicate that IL-2 production, primarily derived from HBV-specific CD4 T cells, was a better measure of viral control than IFN-γ. Furthermore, IL-2 was preferentially enhanced by PD-1 blockade, and this enhancement of T cell function correlated with the IRC assay known to predict outcomes in patients with cancer.

The challenge associated with developing immune biomarkers for HBV is largely related to the difficulty of measuring the magnitude and functionality of HBV-specific immunity, our best correlate of viral control. The frequency of HBV-specific T cells is incredibly low, and the assays to measure the T cell response are complex and complicated by patient variability. Furthermore, analysis of HBV-specific T cell functionality *ex vivo* has been largely restricted to IFN-γ production because of its antiviral activity against HBV. However, addition of IL-2 using the fluorospot assay suggests it is superior to IFN-γ in relation to viral control. *Ex vivo* intracellular cytokine staining validated that a majority of IL-2 is produced by CD4 T cells, providing further evidence for the key role CD4 T cell play in achieving functional cure in patients with CHB.^7,9^

In an ideal situation, we would validate that increased IL-2 responsiveness *ex vivo* following PD-1 blockade would correlate/predict outcomes in patients with CHB being treated with PD-1/PD-L1 targeting drugs. However, because these drugs are in phase 1, or early phase 2 for CHB therapy, patient cohorts to validate our observation are not accessible. Therefore, we used the IRC, which can predict response to PD-1 therapy, to further support the predictive capacity of IL-2. We showed a strong correlation between the IRC and restoration of T cell functionality after PD-1 blockade in the fluorospot assay. Importantly, a pre-existing type I IFN signature in the PBMC did not impact response to IFN-β in the assay. NRs had the highest ISP expression *ex vivo* (Figure 7D) and then showed the greatest upregulation of ISG15 after IFN-β expression (Figure 7E), suggesting the assay is widely applicable, even in the heterogeneous patient population with CHB where type I IFNs are detectable in the periphery in patients with elevated ALT.^41^

Use of a flow cytometry/CyTOF-based assay to predict outcomes provides multiple advantages over measuring the HBV-specific T cell response. There is no need for complicated peptide pulsing protocols, just an overnight stimulation with IFN-β. Detection of ISP-positive cells is well above the sensitivity of the assay rather than a frequency of HBV-specific T cells that often hovers just above assay noise. The IRC assay requires less than 5 mL of blood compared to the 20+ mL needed to test the total HBV-specific T cell response. The development and validation of such an assay could see immunological biomarkers deployed in clinical labs without the need for a specialized immunology lab. Furthermore, a peripheral assay that can enrich for responsive patients would be useful in selecting patients for translational studies that could refine therapeutic strategies. Fine-needle aspirates provide the opportunity to longitudinally sample the liver, but they also make enrollment challenging, limiting sample size, which becomes a significant issue if only 20%–30% of patients see efficacy.

With respect to the T cell profile, we detected HBV-specific T cells *ex vivo* across all CHB cohorts. More IL-2+ HBV-specific T cells were found in patients with lower viral load, such as IC and FC patients, while IFN-γ+ T cell counts did not differ between CHB phases. Using ex vivo intracellular cytokine staining, we confirmed that the majority of IL-2 was produced by CD4 T cells. While not significant, there was a higher trend in IL-2+ CD4 T cell frequency in patients with HBeAg− disease, supporting a role for CD4 T cells in HBV control. Overall, HBV-specific T cell hierarchy followed Core>Pol>Env>X. Core- and Pol-specific responses may be found at similar frequencies, but Env- and X-specific T cells were distinctly weaker.^14,19,20,35,42^ However, this hierarchy diverged between IT and IC patients, similar to previous studies.^3,43^ HBeAg+ patients had significantly fewer Core-specific T cells compared to HBeAg− patients, further supporting the fact that the HBV-specific T cell repertoire is shaped by the natural history of disease.

The frequency of Env-specific T cells was equivalent between IT and IC patients. At first, this appeared to contrast recent studies, which demonstrated that IFN-γ+ Env-specific responses detected after *in vitro* expansion waned in older IC patients.^35,42^ However, when calculated as a proportion of the overall HBV-specific response, Env-specific responses were predominant in IT patients because of lesser Core and Pol-specific responses. These data suggest that Env-specific T cells proliferate poorly during in vitro expansion studies but are functionally detectable *ex vivo*. This highlights important differences when analyzing T cell data obtained from *ex vivo* versus *in vitro* expansion analysis.

The primary goal of checkpoint inhibitor therapy is to enhance the functionality of HBV-specific T cell responses. The IC cohort displayed robust restoration for both IFN-γ and IL-2 after PD-1 blockade. In contrast, IA+ patients exhibited minimal restoration. Functional restoration among the IA− cohort indicates that liver inflammation alone does not account for the poor levels of restoration observed among IA+ patients. However, the T cell antigen hierarchy data provide insight into this discrepancy between cohorts. Core-specific T cells showed the most significant restoration of IL-2 production after PD-1 blockade in IC patients. The presence of HBeAg in IT patients significantly reduced the frequency of Core-specific T cells, which could not be restored with PD-1 blockade. Therefore, functional restoration was weakest in IT and IA+ patients who were both HBeAg+. The impact of HBeAg on Core-specific T cells would also impact correlations with HBsAg. The patients with higher HBsAg levels were IT and IA+ patients. As such, we found a negative correlation between HBsAg levels and IL-2 restoration (not shown). However, because of reduced Core-specific T cells in HBeAg+ patients, the negative correlation was independent of HBsAg, particularly because the frequency of HBsAg-specific T cells was unchanged between IT and IC patients. The interpretation is complex but suggests that patients with low HBsAg (HBeAg−) may benefit from nucleoside analog withdrawal, or novel therapies, because of functional Core-specific T cells.^8^

IC patients also displayed the highest expression of PD-L1 in liver biopsies. The high expression of PD-L1 in IC patient biopsies was counterintuitive. While inflammation appeared to drive PD-L1 expression on immune cells in patients with hepatitis, PD-L1 expression was elevated on both immune cells and hepatocytes in IC patients, indicating greater overall expression in IC patients. Our dataset was not able to determine if PD-L1 expression was related to HBsAg levels, the natural history of CHB, or patient age. However, we hypothesize that expression of PD-L1 in IC patient livers may be responsible for maintaining T cell functionality by restricting constant activation in the liver and suggests checkpoint inhibitor therapy may be sufficient to push, at least a proportion of these patients, to functional cure. It remains to be determined whether the risk:benefit of PD-1 targeting therapies in IC patients, with well-controlled disease, is justified given the potential for significant immune-related adverse events associated with checkpoint inhibitors.

Overall, we demonstrated detectable ex vivo HBV-specific responses across all phases of CHB. The degree of functional T cell restoration could be quantified to potentially stratify treatment-responsive patients for α-PD-1 therapy, which was consistent with the IRC assay. HBeAg− patients were highly responsive to PD-1 blockade, having both significantly improved IFN-γ+ and IL-2+ HBV-specific T cell responses. We showed differential antigen-specific hierarchies between IT and IC patients and that functional restoration was dependent on the HBV antigen targeted. While these findings advance progress toward potentially selecting patients for PD-1 therapy, *ex vivo* FluoroSpot assays, and the necessary HBV OLP, are not widely available in clinical settings. Therefore, validating the IRC approach in PBMC from patients with CHB prior to treatment may provide important predictive ability to predict patient responsiveness to immunotherapies.

## Supplementary Material
