# Supplementary material for: IL-2 produced by HBV-specific T cells as a biomarker of viral control and predictor of response to PD-1 therapy across clinical phases of chronic hepatitis B
Source: Hepatol Commun. 2023 Dec 7;7(12):e0337. doi: 10.1097/HC9.0000000000000337 (PMC10984660; doi:10.1097/HC9.0000000000000337)

Figure 1. Ex vivo detection of HBV-specific T cells in CHB patient cohorts via multi-analyte FluoroSpot assays

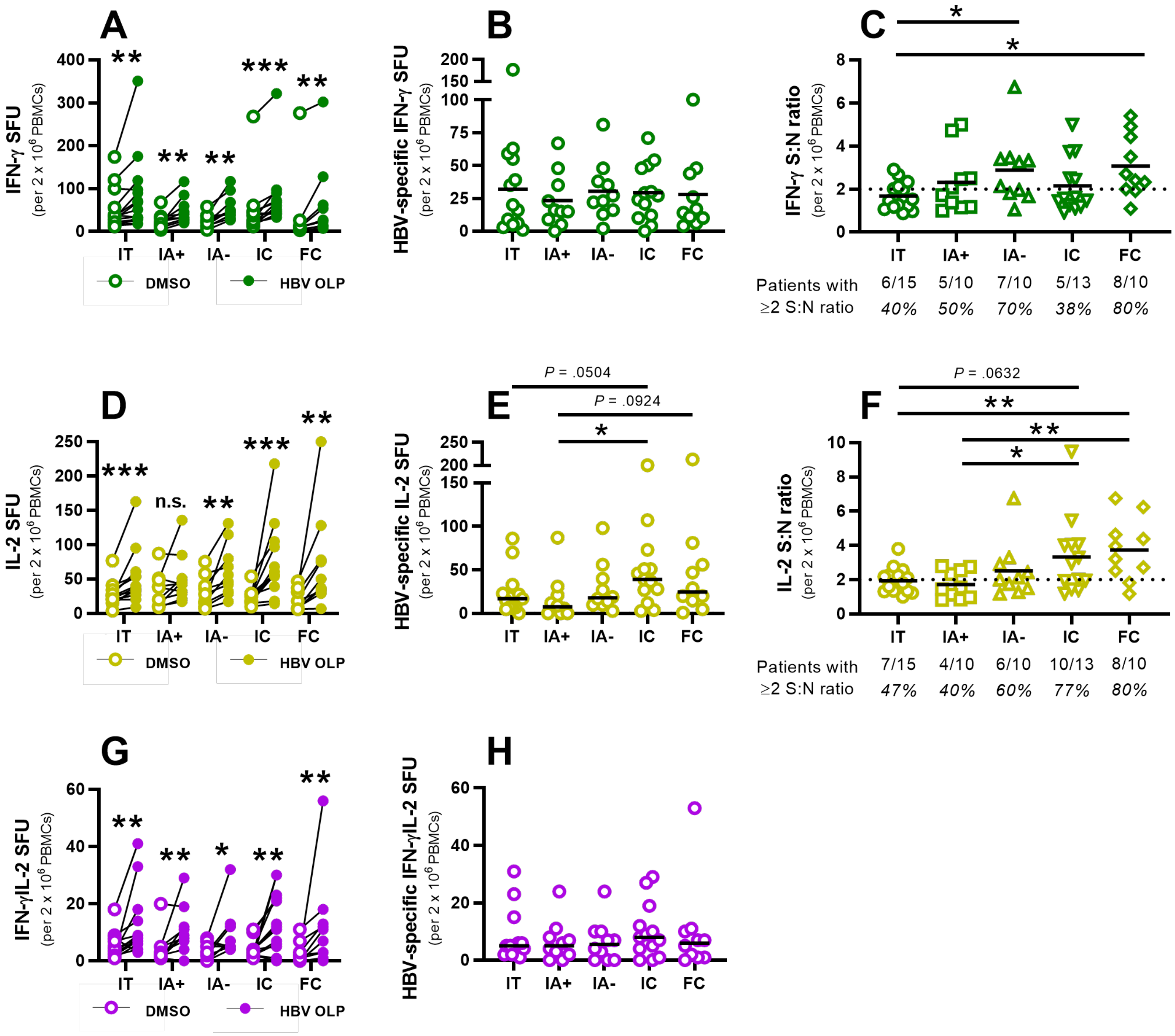

Figure 2. Ex vivo α-PD-1 blocking leads to functional restoration of HBV-specific T cells among CHB patients

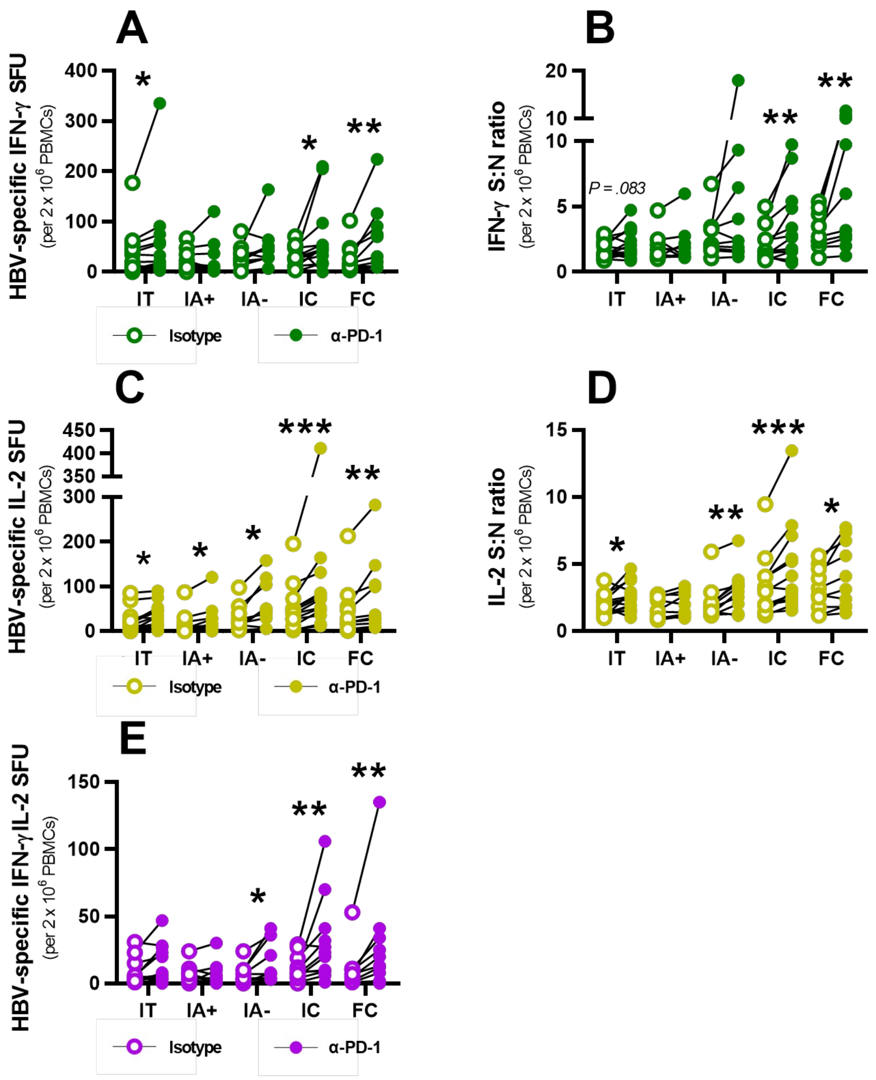

Figure 3. Functional restoration from ex vivo PD-1 blockade across CHB patient cohorts.

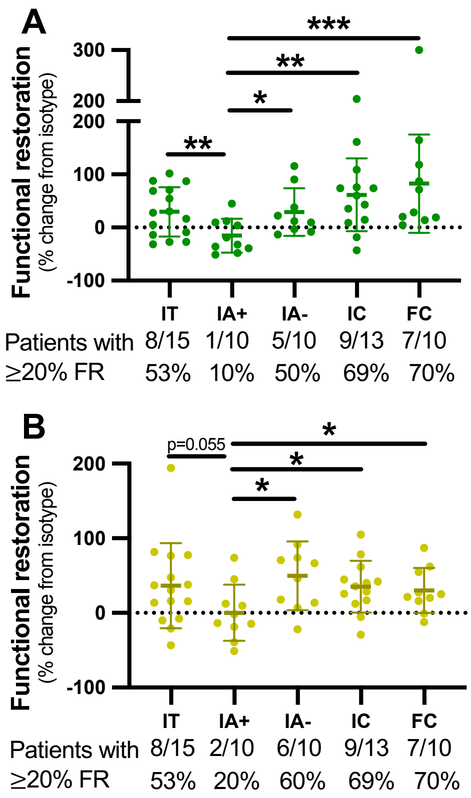

Figure 4. Liver biopsy stains for PD-1/PD-L1 across CHB patient cohorts.

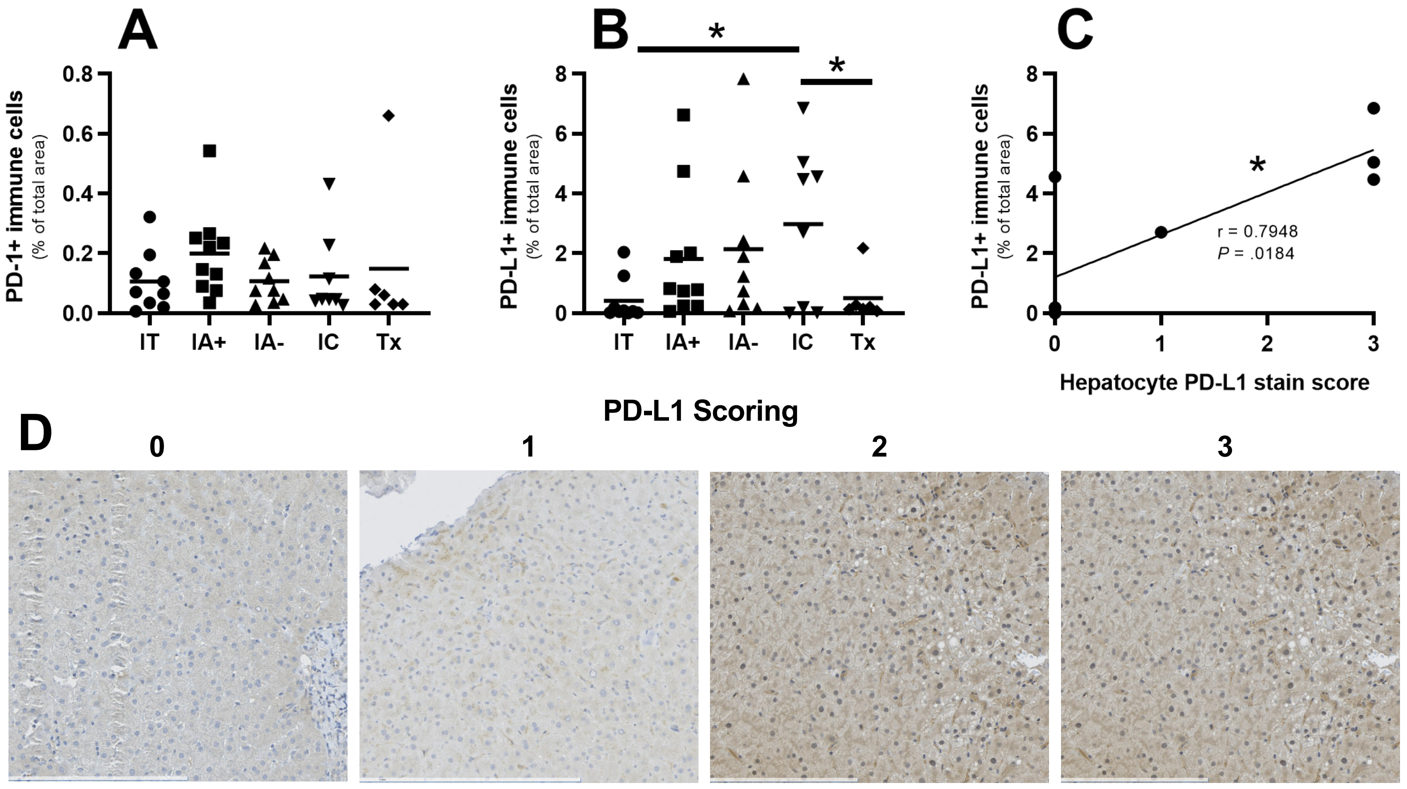

Figure 5. Ex vivo HBV antigen hierarchies are distinct between CHB phase

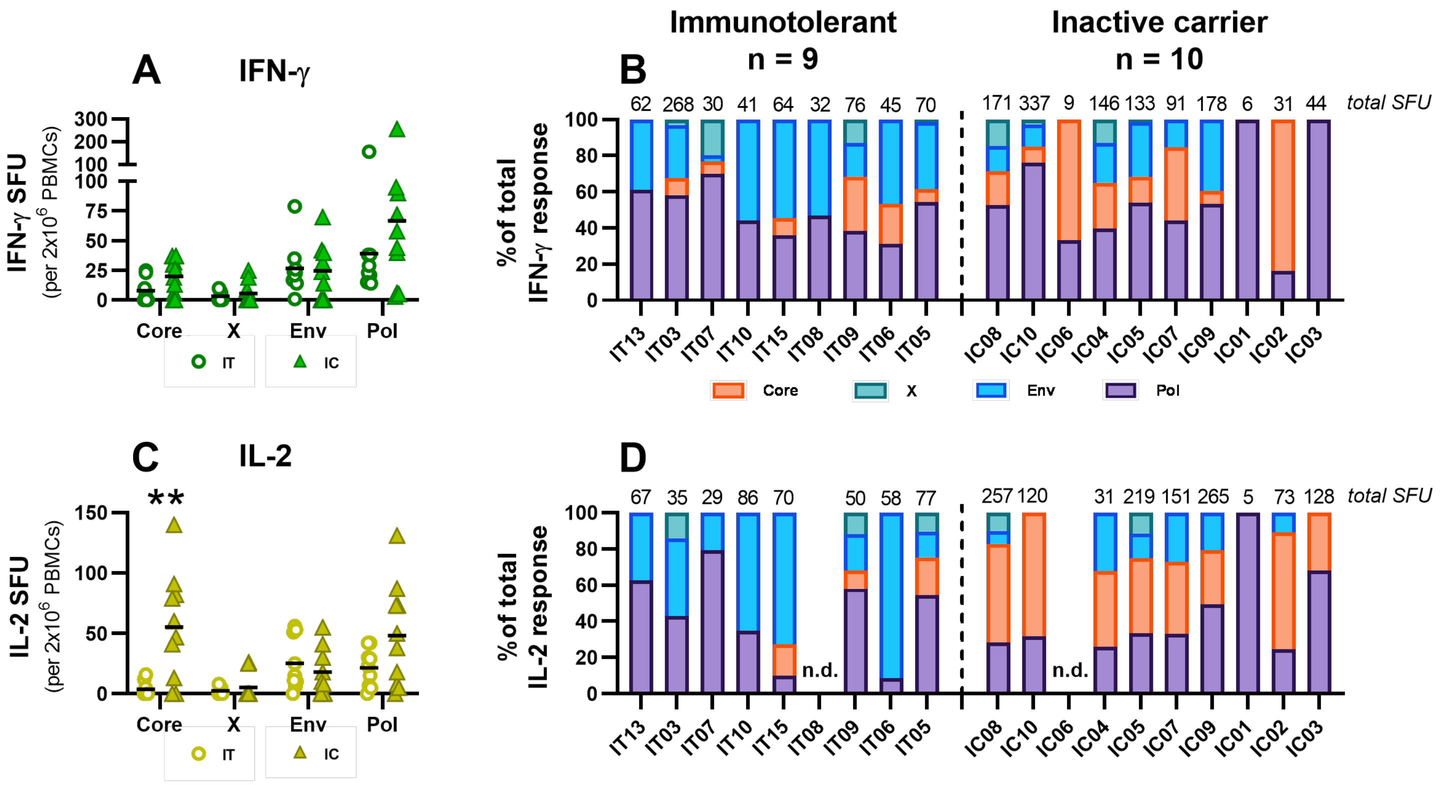

Figure 6. Differential functional restoration of HBV antigen-specific T cell responses by clinical phase and cytokine response

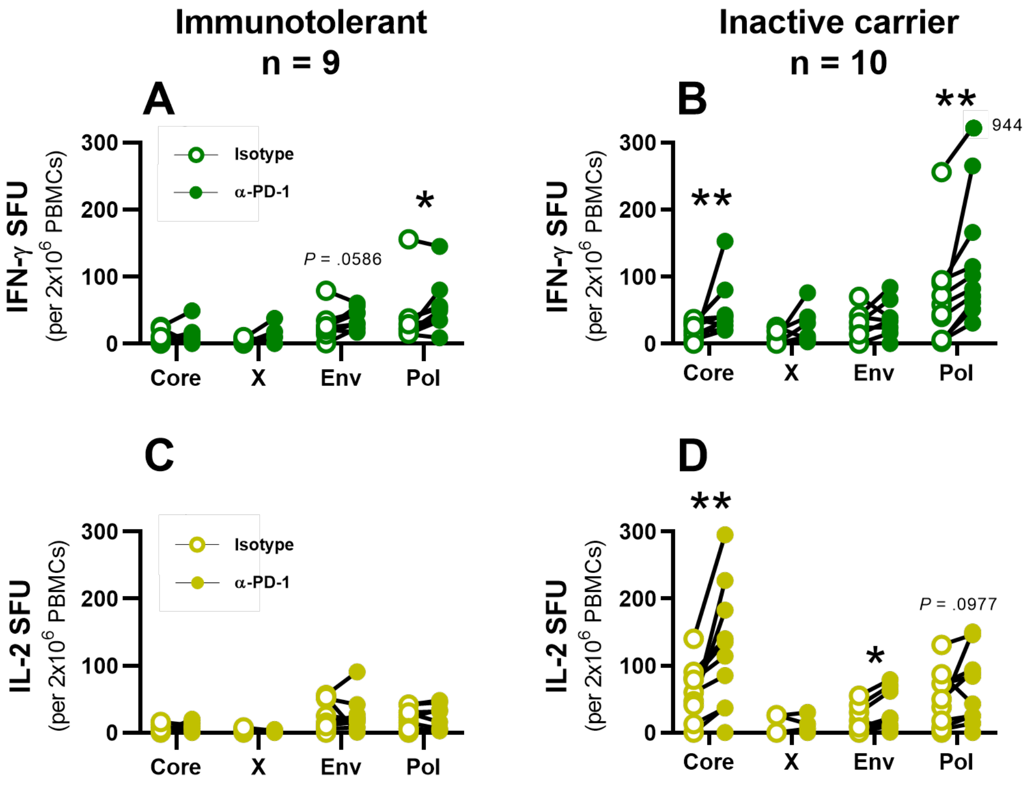

Figure 7. IFN response capacity correlates with IL-2 restoration after PD-1 blockade

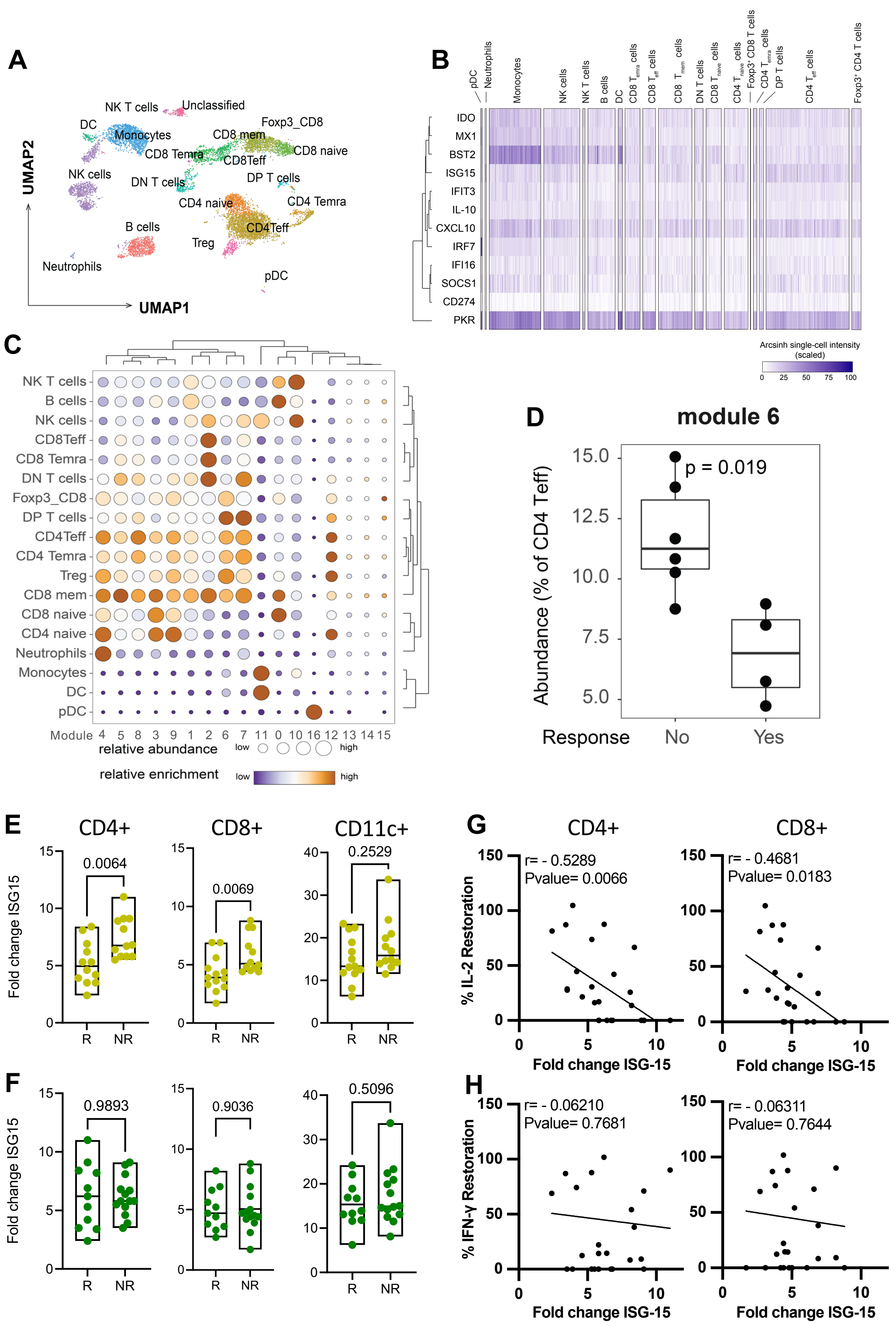

Supplementary Table 1. Chronic HBV patient PBMC cohort characteristics:

Variables	IT	IA+	IA-	IC	FC
Number of patients	15	10	10	13	10
Age (year), median (IQR)	41 (36 - 51)	32 (28 - 39)	41 (37 - 51)	50 (43 - 56)	62 (57 - 66)
Males (Females)	3 (12)	7 (3)	5 (5)	6 (7)	4 (6)
HBsAg (log IU/mL), median (IQR)	4.91 (4.75 – 5.00)	4.62 (4.00 – 4.96)	3.39 (3.02 – 3.62)	3.04 (2.16 – 3.68)	-
HBV DNA (log IU/mL), median (IQR)	8.42 (8.23 – 8.72)	8.28 (7.67 – 8.54)	6.00 (5.64 – 7.28)	1.87 (1.00 – 2.82)	-
ALT (U/L), median (IQR)	24 (22 – 31)	73 (67 - 102)	102 (74.5 – 304)	24.5 (19 – 27)	22 (19 - 24)

IT: Immunotolerant; **IA+:** HBeAg+ immune-active disease; **IA–:** HBeAg– immune-active disease; **IC:** inactive carrier; **FC:** functionally cured

Supplementary Table 2. Chronic HBV patient Biopsy cohort characteristics:

Variables	IT	IA+	IA-	IC	Tx
Number of patients	9	10	9	8	6
Age (year), median (IQR)	32 (26 - 36)	39 (28 - 49)	34 (30 - 50)	41 (37 - 42)	45 (39 - 59)
Males (Females)	5 (4)	7 (3)	6 (3)	6 (2)	5 (1)
HBV DNA (log IU/mL), median (IQR)	8.6 (8.3 – 8.8)	8.2 (7.5 – 8.4)	5.7 (5.3 – 7.0)	2.1 (1.4 – 2.5)	2.7 (2.1 – 3.0)
ALT (U/L), median (IQR)	31 (21 - 38)	148 (90 - 175)	131 (106 - 180)	27 (24 - 36)	31 (23 - 60)

IT: Immunotolerant; **IA+:** HBeAg+ immune-active disease; **IA–:** HBeAg– immune-active disease; **IC:** inactive carrier; **Tx:** on antiviral treatment

Supplementary figure 1. DMSO background was not significantly different upon PD-1 blockade

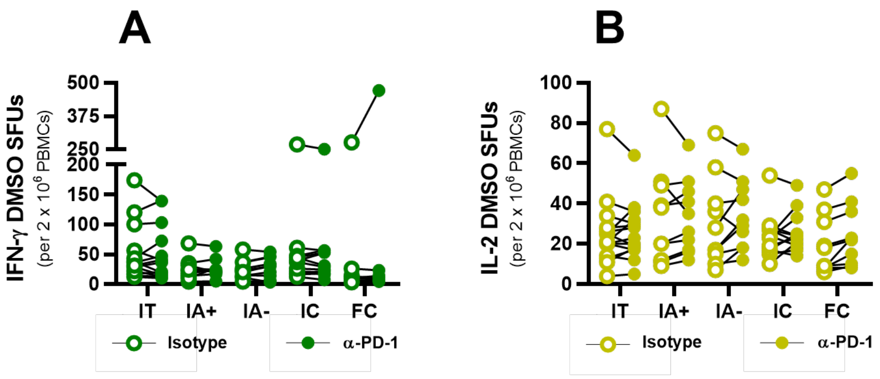

Supplementary figure 2. Env-specific T cell frequencies do not correlate with patient age

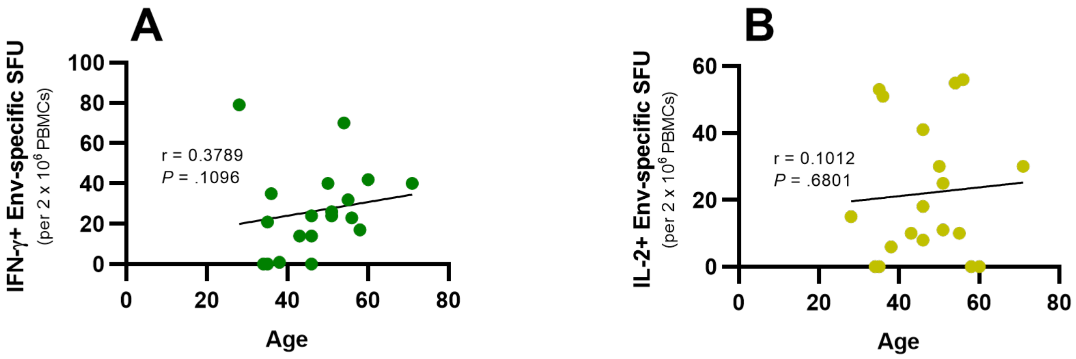

Supplementary figure 3. Ex vivo intracellular cytokine staining for IL-2 in HBV-specific CD4 and CD8 T cells.

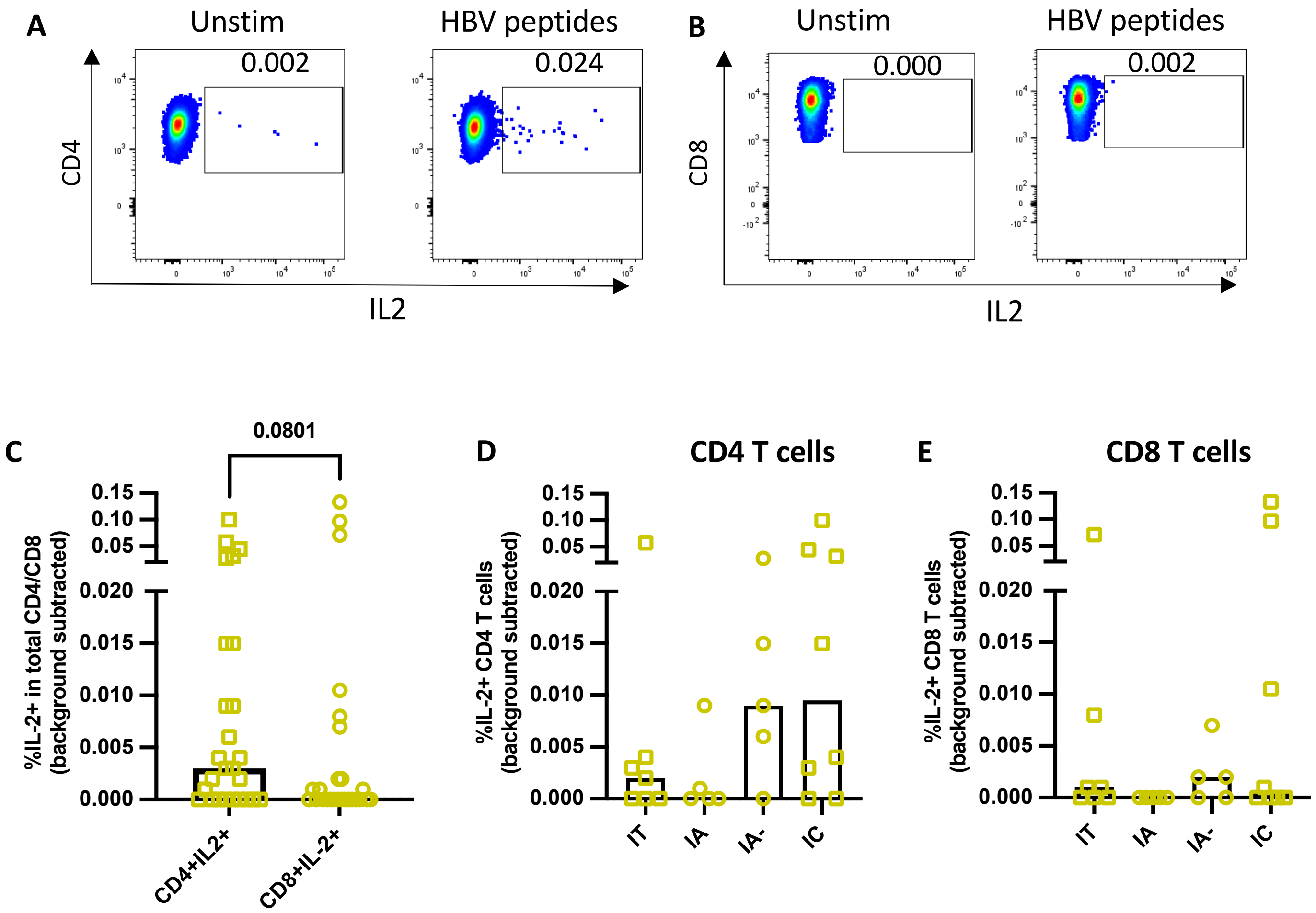

Supplemental Fig 4. ISP modules and expression between CHB patients with increased IL-2 response vs. non-responders

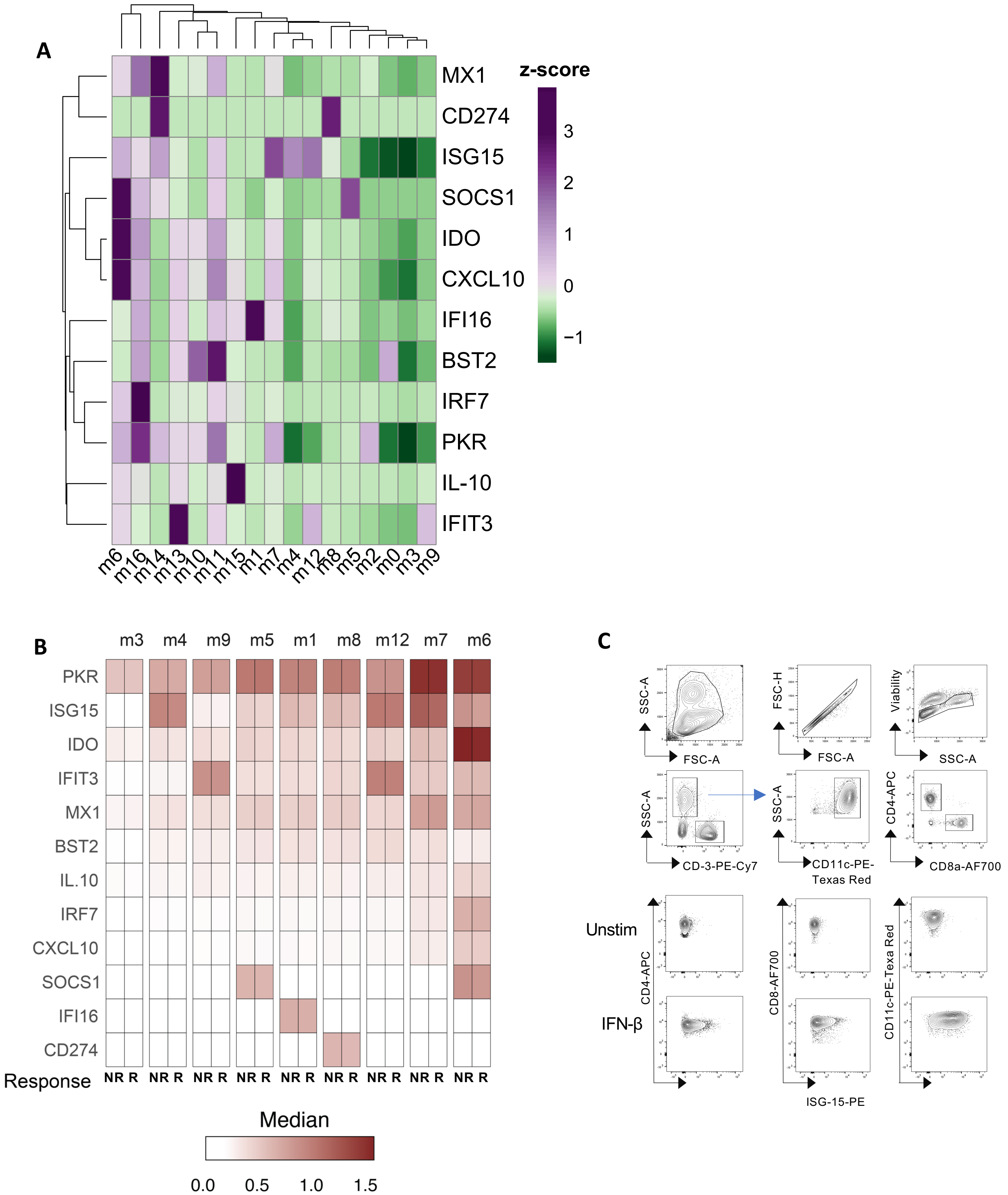

Supplement: Supplementary file 1 [file hc9-7-e0337-s001.pdf]
